# Profile of persons placed under guardianship in the psychiatric department of Monastir

**DOI:** 10.1192/j.eurpsy.2023.1864

**Published:** 2023-07-19

**Authors:** B. Amamou, I. Betbout, M. Ben Mbarek, A. Ben Haouala, F. Zaafrane, L. Gaha

**Affiliations:** Psyciatry, faculty of medicine of Monastir, university of monastir, Monastir, Tunisia

## Abstract

**Introduction:**

The legally incapacitated major is any person who, having acquired the legal majority, should therefore enjoy his rights, and face his social duties, but because of an alteration of his physical or mental faculties, is not able to provide alone for the safeguarding of his interests, nor to face alone his social obligations; there is, therefore, a need for protection which is the placing under guardianship.

**Objectives:**

To describe the clinical and sociodemographic profile of the subjects put under guardianship in the psychiatric services of Monastir.

**Methods:**

This is a retrospective descriptive study that focused on 71 files of subjects examined in the context of psychiatric expertise of guardianship in the psychiatric department Fattouma Bourguiba of Monastir during the period from 10-02-2016 to 08-06-2022.

**Results:**

In total, we included 71 files of the subjects who were examined in the framework of the expert assessments of guardianship. The average age was 53 years. The predominance of males was noted with a sex ratio M/F = 1.5. Most of the patients were of urban origin; more than half (54.9%) were single with an average socioeconomic level for most of the patients (81.7%); 53.5% were illiterate and only 11.3% had a higher education level.

Among the somatic antecedents in our population, 40.9% had neurological pathologies: stroke 8.5%, epilepsy 7%, and dementia 9.9%. The psychiatric history (71.8%) was marked by intellectual disability (47.9%) followed by schizophrenia (28.2%).

Neuropsychiatric comorbidity was noted in 25.4%. The diagnoses retained at the time of the expertise were: intellectual disability (47%), followed by psychosis and dementia with similar percentages of 21%. The duration of the evolution of the symptoms in our population varied between 1 year and 60 years with an average of 22.56 years.

The request for guardianship was made by the siblings in 40.8% of cases, followed by the ascendants in 21.1%.

All the assessments took place at the hospital on a pre-arranged appointment and were formulated in French language.

**Conclusions:**

The knowledge of the specificities of the different Tunisian laws governing guardianship is essential and the meticulous drafting of expert reports requires adapted training, which should be included in the basic training modules for psychiatrists.

**Disclosure of Interest:**

None Declared

